# New approaches to determine fatigue in elite athletes during intensified training: Resting metabolic rate and pacing profile

**DOI:** 10.1371/journal.pone.0173807

**Published:** 2017-03-15

**Authors:** Amy L. Woods, Laura A. Garvican-Lewis, Bronwen Lundy, Anthony J. Rice, Kevin G. Thompson

**Affiliations:** 1 Research Institute for Sport and Exercise, University of Canberra, Bruce ACT, Australia; 2 Australian Institute of Sport, Bruce ACT, Australia; 3 Mary Mackillop Institute for Health Research, Australian Catholic University, Melbourne, Victoria, Australia; University of Rome, ITALY

## Abstract

**Background:**

Elite rowers complete a high volume of training across a number of modalities to prepare for competition, including periods of intensified load, which may lead to fatigue and short-term performance decrements. As yet, the influence of substantial fatigue on resting metabolic rate (RMR) and exercise regulation (pacing), and their subsequent utility as monitoring parameters, has not been explicitly investigated in elite endurance athletes.

**Method:**

Ten National-level rowers completed a four-week period of intensified training. RMR, body composition and energy intake were assessed PRE and POST the four-week period using indirect calorimetry, Dual-Energy X-Ray Densitometry (DXA), and three-day food diary, respectively. On-water rowing performance and pacing strategy was evaluated from 5 km time trials. Wellness was assessed weekly using the Multicomponent Training Distress Scale (MTDS).

**Results:**

Significant decreases in absolute (mean ± SD of difference, p-value: -466 ± 488 kJ.day^-1^, p = 0.01) and relative RMR (-8.0 ± 8.1 kJ.kg.FFM^-1^, p = 0.01) were observed. Significant reductions in body mass (-1.6 ± 1.3 kg, p = 0.003) and fat mass (-2.2 ± 1.2 kg, p = 0.0001) were detected, while energy intake was unchanged. On-water 5 km rowing performance worsened (p < 0.05) and an altered pacing strategy was evident. Fatigue and total mood disturbance significantly increased across the cycle (p < 0.05), and trends were observed for reduced vigour and increased sleep disturbance (p < 0.1).

**Conclusion:**

Four weeks of heavy training decreased RMR and body composition variables in elite rowers and induced substantial fatigue, likely related to an imbalance between energy intake and output. This study demonstrates that highly experienced athletes do not necessarily select the correct energy intake during periods of intensified training, and this can be assessed by reductions in RMR and body composition. The shortfall in energy availability likely affected recovery from training and altered 5 km time trial pacing strategy, resulting in reduced performance.

## Introduction

Preparation for rowing competition involves a high volume of training across a number of different modalities [1]. Successful training programs often involve periods of overload in order to enhance performance following adequate recovery. An imbalance between training stress and recovery, however, can lead to an abnormal training response and possibly, a state of overreaching. Functional overreaching is characterised by short-term performance decrements, and may be accompanied by psychological and physiological symptoms including mood disturbance, which typically resolve within several days or weeks [2, 3]. Progression of these symptoms may lead to extreme or non-functional overreaching, and at worst, a state of overtraining which may take several months or years for recovery [2–4]. Careful monitoring and periodization of training is therefore required to ensure athletes remain consistent in their preparation, and minimize the risk of maladaptation to training, illness and injury that may be associated with an intensified load.

Sufficient energy intake is also critical for training consistency, since prolonged energy restriction can lead to impaired physiological function, and increased risk of fatigue, ill health and underperformance [5, 6]. Given the typically large training volume of elite rowers (24–38 hours per week), monitoring the load undertaken is vital to minimize the risk of adverse health effects. Equally important, however, is ensuring that adequate energy is available to support training demands and optimal adaptation. Resting metabolic rate (RMR) is the minimum amount of energy the body requires to perform its basic functions at rest [7]. RMR can be used as an indicator of energy availability [8], which is defined as the energy remaining for metabolic processes once energy expenditure has been subtracted from energy intake [8]. Whilst low energy availability is often linked with female athletes in weight-category or aesthetic sports, recent research has found male athletes to be susceptible to similar adverse health effects associated with energy restriction [9]. Furthermore, it is plausible that athletes without deliberately restrictive behaviours may suffer energy restriction simply due to a mismatch between energy intake and expenditure as a result of increased training load, inadvertently putting their health, training adaptation and performance at risk. RMR (and subsequently, energy availability) is predominantly affected by body composition and physical activity, but the influence of any specific training periods remains unclear.

While it is known that energy expenditure during physical activity increases in proportion to the amount of work completed [10], the notion of whether such changes persist at rest is uncertain. There is evidence for acute changes in RMR post-exercise [11–14], but longer-term effects appear, to this point, equivocal. Moderate activity protocols for ≥ 12 weeks have demonstrated increases in RMR in overweight and obese populations [15, 16], and a tendency towards higher RMR with higher training volume in endurance-trained male cyclists has been reported [17]. There is also evidence, however, of stability in RMR following either high-intensity resistance or endurance training in healthy males [18]. However RMR has also been demonstrated to decrease in males and females undertaking endurance training for a marathon [19], potentially due to a compensatory response to intensity or insufficient energy intake [20]. As a result, further research is warranted to explore changes in RMR that may result from consecutive weeks of intensified training in elite athletic populations.

Previous literature [21, 22] has demonstrated that glycogen loading and the level of acute fatigue, when altered prior to exercise, can affect the pacing strategy during a subsequent time trial. However to the authors’ knowledge, the effect of an intensified micro-cycle of training on the pacing strategy during a time trial has not been previously investigated. An athlete’s pacing strategy is thought to be a composite of prior training experience, knowledge of the end-point of the exercise and afferent sensory feedback during the exercise informing a teleoanticipatory and feedforward response [23–25]. In addition, the influence of perceptual responses, motivation and decision making (assessing the risk versus the benefit of the exercise), have also been proposed to affect exercise regulation [26]. Clearly, a chronic period of intensified training leading toward a fatigued or even overreached state would present a significant challenge with regard to exercise regulation during a time trial, yet to date this has not been evaluated.

The aim of the present study was to determine whether four weeks of intensified training influences RMR and exercise regulation in elite rowers. We hypothesized that the training block would decrease athletes’ RMR and lead to a more conservative pacing strategy in time trials due to residual fatigue. Periods of heavy training are commonly utilized to promote physiological adaptation and performance enhancement following sufficient recovery, so it is necessary to investigate the metabolic demands of these conditions. Monitoring changes in RMR and exercise regulation during a period of intensified training might enhance understanding of the effects of heavy blocks of training used to prepare athletes for optimal performance.

## Materials and methods

### Study design

Seventeen elite rowers were recruited to undertake four weeks of intensified training at the Reinhold Batschi National Training Centre, Canberra, Australia. The study was approved by the Australian Institute of Sport Human Ethics Committee and University of Canberra Human Research Ethics Committee according to the Declaration of Helsinki. All athletes provided written informed consent prior to involvement. The four-week training cycle included a combination of on-water, ergometer, strength and cross-training sessions for six days per week. RMR, body composition and rowing pacing strategy were assessed PRE and POST the four-week block. Wellness and training sessions were monitored weekly.

### Participants

Seventeen male (n = 10) and female (n = 7) rowers aged 21–30 years participated in the study. All athletes had nominated for selection to the 2015 Australian Rowing Team. Mean ± standard deviation (SD) height and body mass of the group was 186.0 ± 7.8 cm and 80.8 ± 12.5 kg, respectively. All athletes achieved similarly high levels of physical readiness prior to the study beginning, as data collection occurred three months into the 2015 domestic season.

### Training load

Training load was assessed in T2 minutes; a validated unit of training load utilized within the Rowing Australia high performance network [27]. The T2-minute calculation incorporates training duration, intensity, and mode to provide a consistent system for quantifying loads from varied training formats within the elite-rowing program. One T2 minute is equivalent to one minute of on-water single scull rowing at T2 intensity (∼60–72% VO_2_max) [28]. Training load for the week prior to the study (PRE) was 1490 ± 390 T2 minutes, which increased to 1907 ± 155 (+28%), 1861 ± 208 (+25%), 1762 ± 92 (+18%) and 1664 ± 120 T2 (+12%) minutes for weeks 1, 2, 3 and 4 of the training period; and decreased to 1141 ± 177 (-23%) T2 minutes for the week following its completion (POST).

### Resting metabolic rate

RMR was measured four days prior to and following completion of the training period using the Douglas Bag method of indirect calorimetry, which has been described previously [29, 30]. Briefly, athletes presented to the laboratory between 0500 and 0900, and rested supine for 25 minutes prior to testing. The Douglas Bag measurement of RMR involves collecting expired air through a one-way mouthpiece into gas-impermeable collection bags, and subsequently analysing the expirate with high-precision oxygen (O_2_) and carbon dioxide (CO_2_) analysers. Gas volume is measured using a water-sealed Tissot Spirometer; while Haldane transformations for the calculation of inspired-to-expired volume conversions allow for the calculation of minute O_2_ consumption and CO_2_ production [31], which are converted to kilojoule equivalents based on standard formulas [31, 32]. All athletes were overnight rested and fasted, and abstained from physical activity for at least eight hours prior to all measurements. In the present applied setting, it was not possible to assess the athletes following a rest day and as such, both RMR measurements were conducted in the morning following an afternoon strength training session. Minute ventilation (V_E(STPD)_] was assessed for each expirate collection. Typical error (TE) for the Douglas Bag method of RMR measurement in our hands is 286.8 kJ, or 4.3% [90% confidence limits (CL): 3.1–7.2%] within days, and 455.3 kJ or 6.6% (90% CL: 4.8–11.1%) between days, which compares favourably with other researchers [10].

### Body composition and energy intake

Body composition was assessed immediately following each RMR measurement via Dual-Energy X-Ray Densitometry (DXA; GE Lunar Prodigy, GE Healthcare Asia-Pacific). Each DXA scan provided an assessment of fat mass, lean mass and bone mineral content (BMC). Fat-free mass (FFM) was calculated as lean mass plus BMC. Radiation safety approval was provided by the Radiation Safety Committee at the John James Hospital, Canberra. Athletes provided a urine sample at first void for assessment of hydration status via urine-specific gravity from digital hand-held refractometer (ATAGO, USA). Energy intake and consumption of macronutrients were recorded for the three days immediately prior to each RMR measurement, and later analysed for total energy and macronutrient intake by an accredited practising dietician using FoodWorks Professional v7.0.3016 (Xyris Software Pty Ltd, Australia). Athletes were not instructed to adhere to certain dietary guidelines or practices in order to assess natural behaviours in an applied setting.

### Rowing performance and pacing strategy

On-water rowing pacing strategy and performance were evaluated using a 5 km time trial in the week prior to, and at the end of the final week of the training cycle as part of Australian Rowing Team selection requirements. Athletes were trialled individually in single sculls using a ‘handicapped’ format, whereby the slowest athlete began their trial first, and the remaining athletes began at 30 s intervals thereafter. Prescribed training was standardized for the two days prior to both time trials. Split time per 500 m, velocity and stroke rate data were obtained using boat-mounted GPS units (Catapult Sports, VIC, Australia) for each trial. RPE was unable to be assessed due logistical constraints and the time-delay between the completion of the trial and the athletes’ return to the boatsheds. Typical error (TE) for on-water rowing performance tests have been reported to be between approximately 1–4% [33]. Logistically, the trials were held in two different locations (PRE: Nepean River, Penrith, NSW; POST: Lake Burley Griffin, Canberra, ACT) due to the training commitments of the athlete group at each time point. Pre-race conditions were assessed for both trials by analysing GPS and accelerometer data immediately prior to the race start. This means that, whilst sitting idle, boat velocity provided an indication of the potential influence of water current, flow, and wind, and the direction of each, on the results achieved. In recognition of the difficulty in comparing the trials due to the possible influence of environmental conditions, data were calculated every 25% of the total race distance and normalised based on average velocity for the trial to allow for an appropriate evaluation.

### Wellness

The Multicomponent Training Distress Scale (MTDS) [34] was administered one week prior (PRE), each week during, and one-week after completion of the training cycle (POST) to assess training-related mood disturbance. Questionnaires were consistently dispensed after breakfast and before the second morning training session on the Friday of each respective training week. Responses to the 22-item questionnaire were anchored on a likert scale from 0 being “Not at all’ to 5 being “Extremely’. Each question corresponded to one of six common indicators of training overload including depressed mood, vigour, physical signs and symptoms, sleep disturbance, perceived stress and fatigue. Responses related to Vigour were negatively coded. The sum of the response scores produced a value for Total Mood Disturbance (TMD).

### Training monitoring

#### On-water training

On-water training sessions were monitored daily for velocity, distance and stroke rate from boat-mounted GPS units. Individual heart rate data from each session was then uploaded to an online software program (Sportlyzer, Tartu, Estonia). Responses to a specified work set (1800 m at 24 strokes-per-minute) were assessed weekly using GPS race time, heart rate, rating of perceived exertion (RPE, 1–10 Borg Scale [35]) and blood lactate concentration (BLa) via earlobe capillary sample (Lactate Pro 2, Arkray, Japan) upon completion.

#### Ergometer training

Ergometer (Model D, Concept 2, Victoria, Australia) sessions were further monitored during weekly 30-minute sets at each athlete’s individual T2 training zone for power output, heart rate, RPE and BLa. Ergometer sessions were completed at a controlled stroke rate of 20 strokes-per-minute for standardization.

### Data analysis

All data satisfied assumptions of normality, sphericity and homogeneity of variance. Differences between PRE and POST for RMR, body composition and performance variables were assessed using paired-samples T-test (POST–PRE), which generated the mean and SD of difference and 95% CL, and percent change. Pacing data within the 5 km time trials were assessed using two-way repeated measures ANOVA [Split (0–25%, 25–50%, 50–75%, 75–100% of race) vs Trial (PRE, POST)], and wellness data was assessed via one-way repeated measures ANOVA. Where significant differences were observed for Tests of Within-Subjects Effects, pairwise comparisons were conducted with the Sidak correction to determine the time course of the difference. Data are presented as mean ± SD with significance set at 0.05 unless otherwise stated.

## Results

### Training load

Weekly training load was increased by (mean ± SD) 21 ± 7% from PRE. Of the initial seventeen athletes recruited, seven athletes were excluded from data analysis due to injury, hyperventilation during RMR measurement (respiratory quotient, RQ > 1.0 [36]) or deliberate manipulation of body composition. Thus, ten athletes were included in the final analysis having completed the training block without incident (n = 5 males, n = 5 females).

### Resting metabolic rate

**Fig 1:**
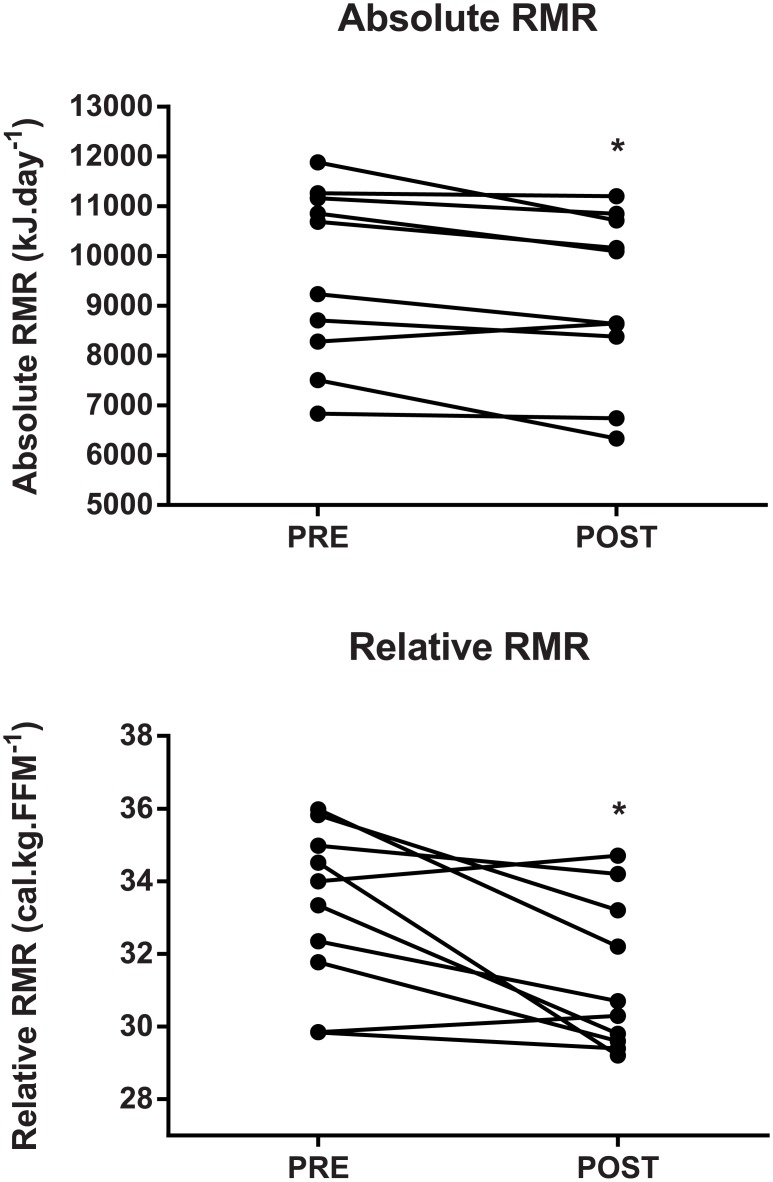
Resting Metabolic Rate (RMR) variables for individual athletes PRE and POST the four-week training cycle. Where a significant difference between time points is observed, * indicates p < 0.05.

Four weeks of intensified training elicited a significant decrease in absolute RMR (mean ± SD of difference, p-value: -466 ± 488 kJ.day^-1^, p = 0.01, Fig 1) and relative RMR (-8.0 ± 8.1 kJ.kg.FFM^-1^, p = 0.01, Table 1).

**Table 1:** RMR and body composition variables PRE and POST the four-week training cycle. Results from paired-samples T-test (POST-PRE) are presented as mean ± SD of difference, 95% CL of difference, and percent change.

Outcome Measure	PRE	POST	Mean ± SD of Difference	95% CL of Difference	P-value	Δ POST-PRE (%)
Absolute RMR (kJ.day^-1^)	9644 ± 1758	9178 ± 1710	-466 ± 488	-815.3 to -116.9	p = 0.01	-4.8
Relative RMR (kJ.kg.FFM^-1^)	139.6 ± 9.4	131.6 ± 8.7	-8.0 ± 8.1	-13.8 to -2.2	p = 0.01	-5.7
Relative RMR (cal.kg.FFM^-1^)	33.2 ± 2.3	31.3 ± 2.1	-1.9 ± 2.0	-3.3 to -0.5	p = 0.01	-5.7
Body mass (kg)	80.8 ± 12.5	79.2 ± 12.9	-1.6 ± 1.3	-2.6 to -0.7	p = 0.003	-2.0
Fat mass (kg)	12.0 ± 5.0	9.8 ± 4.7	-2.2 ± 1.2	-3.1 to -1.3	p = 0.0001	-18.3
Fat-free mass (FFM) (kg)	69.2 ± 13.0	69.9 ± 13.2	0.6 ± 3.4	-1.8 to 3.0	p = 0.57	0.9

### Body composition and energy intake

**Fig 2:**
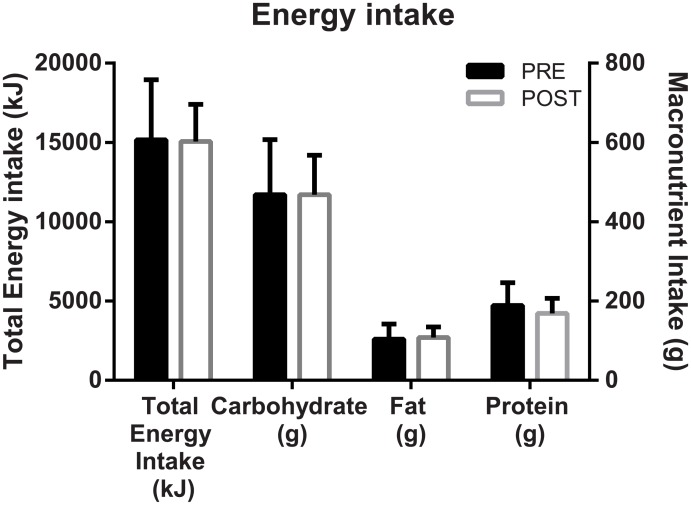
Energy and macronutrient intake PRE and POST the four-week training cycle. Data are presented as mean ± SD for each parameter PRE and POST. Where a significant difference between time points is observed, * indicates p < 0.05.

Significant decreases in body mass (-1.6 ± 1.3 kg, p = 0.003) and fat mass (-2.2 ± 1.2 kg, p = 0.0001) were observed upon completion of the training cycle. FFM was stable (p > 0.05, Table 1). Hydration status via USG was also stable (-0.103 ± 0.580 kg.m^3^, p = 0.81). No differences were observed for total energy intake or individual macronutrients consumed prior to each RMR measurement (Fig 2).

### Rowing performance and pacing strategy

Mean environmental conditions for the PRE trial were (air temperature, wind speed, wind direction, water temperature, elevation) 23.1°C, 3.7 m.s^-1^ north-easterly, 25.4°C, 25 m. Conditions for the POST trial were 11.1°C, 0 m.s^-1^, northerly, 21.7°C, 600 m, respectively. Pre-race conditions for the PRE and POST trials were [mean ± SD boat velocity, direction of travel in degrees, (wind component)] 0.12 ± 0.08 m.s^-1^, 55° (tail), and 0.10 ± 0.05 m.s^-1^, 120–180° (cross-tail), respectively. No differences were observed in the pre-race conditions between time trials (mean ± SD of difference, p-value: -0.01 ± 0.08 m.s^-1^, p = 0.62).

**Fig 3:**
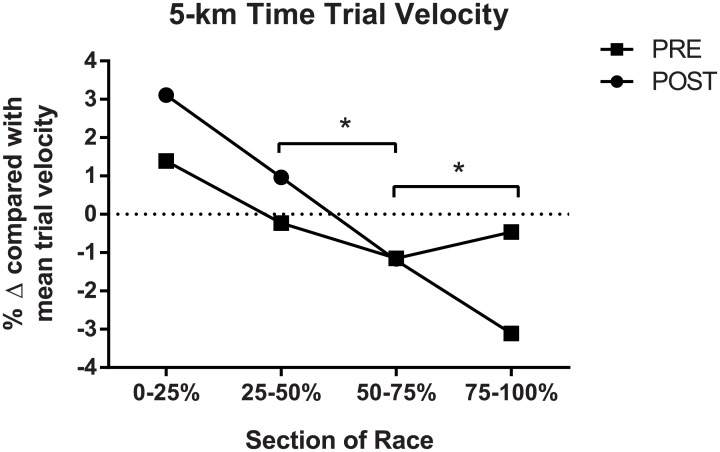
On-water rowing performance via 5 km time trial between PRE and POST. Mean boat velocity for each trial was calculated, with each split (0–25%, 25–50%, 50–75%, 75–100% of race) assessed for percent change compared with the mean. Where a significant interaction between time points is observed, * indicates p < 0.05.

On-water 5 km time trial rowing performance was significantly reduced at the end of the training cycle (p < 0.05). Velocity and stroke rate from each 25% of the races were significantly lower at POST than PRE (p < 0.05, Table 2). Further investigation of the percent change of each split (0–25%, 25–50%, 50–75%, 75–100% of race) compared with the mean boat velocity for each trial revealed a significant interaction between splits 2 and 3 (25–50% and 50–75%: F_(1,8)_ = 16.336, p = 0.004), and splits 3 and 4 (50–75% and 75–100%: F_(1,8)_ = 15.471, p = 0.004), demonstrating a significantly altered pacing strategy at these time points between trials (Fig 3).

**Table 2:** 5 km time trial performance PRE and POST the four-week training cycle. Results from paired-samples T-test (POST-PRE) are presented as mean ± SD of difference and 95% CL of difference.

Outcome Measure	Section of Race	PRE	POST	Mean ± SD of Difference	95% CL of Difference	P-value
Split time per 500 m (mm:ss)	0–25%	1:54.3 ± 00:07.9	1:56.7 ± 00:08.1	0:02.4 ± 0:02.3	00:00.7 to 00:04.2	p = 0.01
	25–50%	1:56.2 ± 00:08.0	1:58.9 ± 00:08.0	00:02.7 ± 00:00.8	00:02.1 to 00:03.3	p = 0.0001
	50–75%	1:57.3 ± 00:07.6	2:01.4 ± 00:07.4	00:04.1 ± 00:01.5	00:03.0 to 00:05.2	p = 0.0001
	75–100%	1:56.3 ± 00:07.3	2:04.1 ± 00:09.9	00:07.8 ± 00:02.9	00:05.6 to 00:10.0	p = 0.0001
Velocity (m.s^-1^)	0–25%	4.39 ± 0.30	4.31 ± 0.30	-0.09 ± 0.09	-0.15 to -0.02	p = 0.02
	25–50%	4.32 ± 0.29	4.22 ± 0.28	-0.10 ± 0.04	-0.13 to -0.07	p = 0.0001
	50–75%	4.28 ± 0.27	4.13 ± 0.25	-0.14 ± 0.05	-0.19 to -0.10	p = 0.0001
	75–100%	4.31 ± 0.26	4.05 ± 0.32	-0.26 ± 0.07	-0.32 to -0.21	p = 0.0001
Stroke Rate (strokes per minute)	0–25%	32.7 ± 2.0	30.5 ± 1.5	-2.3 ± 0.6	-2.7 to -1.8	p = 0.0001
	25–50%	31.8 ± 1.6	30.0 ± 1.24	-1.8 ± 0.6	-2.3 to -1.4	p = 0.0001
	50–75%	31.9 ± 1.6	30.0 ± 1.3	-2.0 ± 0.5	-2.4 to -1.6	p = 0.0001
	75–100%	32.8 ± 1.8	30.1 ± 2.4	-2.6 ± 1.4	-3.7 to -1.5	p = 0.001

### Wellness

MTDS responses demonstrated significant increases in ‘Fatigue’ (Tests of Within-Subjects Effects: F_(5,45)_ = 5.413, p = 0.001) and ‘Total Mood Disturbance (TMD)’ (F_(5,45)_ = 3.180, p = 0.02) throughout the training cycle (Table 3). Post-hoc analysis failed to reveal the time course of differences (p > 0.05).

**Table 3:** Multi-component Training Distress Score (MTDS) parameters throughout the four-week training cycle. Data are presented as mean ± SD. Results from a one-way repeated measures ANOVA for each component are presented as the F-statistic for Tests of Within-Subjects Effects.

Outcome Measure	PRE	Week 1	Week 2	Week 3	Week 4	POST	F-Statistic
Depressed Mood	0.84 ± 1.04	0.66 ± 0.92	0.76 ± 1.08	0.42 ± 0.51	0.20 ± 0.16	0.56 ± 0.67	F_(5, 45)_ = 1.089, p = 0.38
Vigour	2.20 ± 0.48	2.00 ± 1.10	2.73 ± 0.82	2.53 ± 0.76	1.98 ± 0.56	2.25 ± 0.89	F_(5, 45)_ = 2.307, p = 0.06
Physical Signs and Symptoms	2.33 ± 0.44	2.07 ± 0.87	2.07 ± 0.98	2.57 ± 0.80	1.97 ± 0.79	1.97 ± 0.66	F_(5, 45)_ = 1.233, p = 0.31
Sleep Disturbances	1.50 ± 1.49	1.77 ± 1.38	1.37 ± 1.12	1.23 ± 0.94	0.90 ± 0.93	0.53 ± 0.74	F_(5, 45)_ = 2.049, p = 0.09
Perceived Stress	1.05 ± 1.05	0.65 ± 0.92	0.95 ± 0.67	0.68 ± 0.46	0.53 ± 0.45	0.78 ± 0.70	F_(5, 45)_ = 0.896, p = 0.49
Fatigue	2.03 ± 0.64	2.27 ± 0.81	2.53 ± 0.98	2.23 ± 0.75	1.30 ± 0.81	1.43 ± 0.82	F_(5, 45)_ = 5.413, p = 0.001
Total Mood Disturbance (TMD)	9.96 ± 3.52	9.41 ± 3.52	10.40 ± 3.20	9.65 ± 2.60	6.87 ± 2.50	7.52 ± 2.39	F_(5, 45)_ = 3.180, p = 0.02

### Training monitoring

Insufficient data was obtained for statistical analysis, however descriptive responses to the weekly monitored on-water 1800 m repetition and weekly 30-minute ergometer set (race time, power output, heart rate, RPE, BLa), both at a fixed stroke rate, are presented in Table 4.

**Table 4:** Descriptive data from weekly on-water and ergometer training sets throughout the four-week training cycle. Due to an insufficient sample, data are presented as mean ± SD for race time, heart rate, rating of perceived exertion (RPE) and blood lactate concentration (BLa) during an on-water 1800 m piece at 24 strokes-per-minute; and power output, heart rate, RPE and BLa during a 30-minute ergometer piece at 20 strokes-per-minute.

Outcome Measure	Week 1	Week 2	Week 3	Week 4
*On-water Monitoring Set*: *1800 m*, *R24*				
Race time (mm:ss)	07:08.6 ± 00:25.6	06:49.5 ± 00:13.0	07:02.5 ± 00:10.0	07:16.1 ± 00:24.2
Heart rate (bpm)	177 ± 10	186 ± 1	185 ± 3	176 ± 12
RPE (1–10)	7 ± 1	9 ± 1	8 ± 1	7 ± 2
BLa (mmol.L^-1^)	3.5 ± 1.3	6.3 ± 0.5	6.0 ± 1.6	4.2 ± 2.0
*Ergometer Monitoring Set*: *30-minutes T2*, *R20*				
Power output (W)	271 ± 12	257 ± 32	261 ± 22	235 ± 48
Heart rate (bpm)	163 ± 5	167 ± 5	153 ± 9	149 ± 12
RPE (1–10)	5 ± 1	6 ± 1	5 ± 2	5 ± 1
BLa (mmol.L^-1^)	2.2 ± 0.2	1.7 ± 0.7	1.8 ± 0.4	1.6 ± 0.5

## Discussion

### Main findings

The present study demonstrates that four weeks of intensified training can significantly decrease absolute and relative RMR, body mass and fat mass, and increase fatigue and mood disturbance in elite rowers. These findings may be related to the nature of the training, the load imposed, and a likely decrease in energy availability due to an imbalance between energy intake and expenditure. Secondly, it is also plausible that these psychophysiological disturbances affected the rowers’ pacing strategy and performance during the 5 km time trial at the end of the training period, suggesting they were exercising in a substantially fatigued, and possibly overreached state. However we acknowledge that these findings need to be interpreted with caution given that i) individuals when training intensively can exhibit variable individual responses, and ii) the ideal study design would have included a measurement of RPE and controlled environmental conditions for the performance trials, as well as additional follow-up time trials during the recovery period to confirm and further elucidate the extent of the athletes fatigue. Nonetheless, the present findings suggest that marked changes in RMR, body composition, mood responses and time trial pacing strategy occurred, which suggests that RMR and time trial pacing strategy have potential to be used as part of a test battery of objective markers of fatigue and training distress alongside other widely reported measures [3].

### Exercise and energy expenditure

The effect of exercise on RMR has to this point been unclear. Prior research has demonstrated increases [15–17], decreases [19] or no change [18, 37–39] in RMR with a variety of training protocols. Many of these conflicting findings may be due to the population studied, adherence to the protocol, the exercise modality, and in particular, the interval between cessation of exercise and RMR measurement. The present study used criterion measures to determine changes in RMR and body composition to provide the most accurate assessments possible. In both trained and untrained individuals, energy expenditure can remain substantially elevated above resting levels up to 48 hours post-exercise [14]. An elevated RMR immediately following exercise has been postulated to be related to an excess post-exercise oxygen consumption (EPOC), which may involve a prolonged response between 3–24 hours [40], rather than a true physiological change. Indeed, as an exponential relationship between exercise intensity and EPOC has been reported [40], it is paramount to ensure RMR measurements are conducted following sufficient recovery from previous training, particularly during intensified training phases. In the present study, due to morning training commitments, RMR and DXA measurements were only possible on the athletes’ morning off, and it is reasonable to question whether training completed the day prior to measurement, although standardized, may have influenced RMR results. Pursuant to the EPOC hypothesis, however, RMR would have been expected to increase, rather than decrease as was presently observed. The decrease in RMR would suggest that there was a compensatory response to the intensified training load or insufficient energy intake, or both.

### Exercise and energy balance

We observed an approximate 5% decrease in RMR following intensified endurance training with significant changes in body composition, suggesting an energy imbalance. Despite the increases in training load, the rowers’ energy intake remained unchanged, which is of practical concern since a negative energy balance increases risk of injury, illness and overtraining [41–43]. It is feasible that decreased energy availability, due to an insufficient energy intake across the training cycle, affected the fatigue levels, pacing strategy and on-water rowing performance of the rowers. This is an interesting finding, as it is well known amongst athletes that a substantially increased training load would require a deliberate dietary adjustment, particularly in macronutrients such as carbohydrate [34], to support physical demands. Still, it is possible that a lag exists between the initiation of the increased energy expenditure, and a compensatory increase in appetite in elite athletes when undertaking a period of intensified training.

It is plausible that a balance between energy intake and expenditure could attenuate undesirable changes in RMR by increasing energy availability as well as supporting training demands. An increase in dietary intake not only ensures sufficient consumption of macronutrients, but also essential vitamins and minerals to assist in muscle repair and recovery for the ensuing training sessions. However it appears from the current findings that it may not be possible for elite athletes during a micro-cycle of intensified load, which further heightens energetic demands, to detect an energy imbalance and/or to compensate sufficiently. This lack of adjustment might be due to a lack of sensitivity, possibly because they chronically experience lowered glucose concentrations and a high level of energy expenditure during training. It might also relate to the delayed appetite response, coupled with the increased time demand of training consequently reducing access to food sources. Appropriate education and nutritional interventions are thus critical to support energy balance during intensified training cycles of this nature.

### Possible mechanisms of change in RMR

The underlying mechanisms of change in RMR following exercise remain to be elucidated. Fat free mass is the largest determinant of RMR, accounting for up to 70% of individual RMR variation [20]. As a metabolically active tissue, any change in FFM is likely to affect overall energy expenditure, which is a common conclusion from previous investigations [44]. In the present study, however, this notion was not apparent, as FFM remained stable. Therefore, the decrease in RMR is likely attributed to other mechanisms. Energy balance is primarily controlled by the hypothalamus, of which a number of peptide hormones and cytokines are purported to influence. Leptin, in particular, is a satiety hormone produced in the adipose tissue [45], and has a major influence on appetite and energy homeostasis [1, 46, 47]. Leptin has been suggested as a marker of training stress in male rowers [48], and may decrease following high-volume rowing training [49]. Importantly, there appears to be a hypothalamic link between increased energy expenditure and restricted energy intake. Under conditions of negative energy balance, neuroendocrine function is affected, and can result in decreased leptin concentrations, energy conservation and decreased thermogenesis [50]. It is conceivable that the present decrease in resting metabolism was a protective mechanism from changes in body composition. It was not possible to obtain blood or hormonal profiles in the present investigation, however future research would benefit from their inclusion to provide insight on these mechanisms, as well as an indication of training load stress.

### Rowing performance and pacing strategy

Performance in the 5 km time trials did not improve following a micro-cycle of intensified training, and notably the pacing strategy was observed to be significantly different between PRE and POST trials. Split times for the POST 5 km trial were significantly greater than the PRE trial for the first 2500 m (2.1 and 2.3% respectively) which might indicate that a more conservative pacing strategy was conceived from the outset (as hypothesised), however this could also be due to normal variation in performance as the typical error for on-water tests can be within 1–4% [33]. It is also reasonable to suggest the influence of environmental conditions on performance during on-water time trials might explain these present results. However, the analysis of pre-trial boat velocity demonstrated no differences in water current, flow or wind speed between the two time trials, suggesting an alternative explanation is warranted. Conspicuously, the final two split times of the POST trial were 3.5% and 7.1% greater than the PRE trial, which suggests that the rowers suffered either relatively greater fatigue and/or loss of central motor drive or motivation in the POST trial. Interestingly, the PRE trial demonstrated the typical reverse-J-shape parabolic pacing strategy previously observed in elite rowing [51], where, following the initial acceleration, boat speed decreases slightly before rising again in the final quarter with an ‘end spurt’ [52]. However in the POST-trial, a progressive reduction in boat velocity was observed, which is indicative of a positive pacing strategy. A positive pacing strategy is uncommon during on-water rowing [43] and would suggest either i) an early onset of fatigue occurred which would indicate a pacing error, and/or ii) that a decision was made to exercise at a lowered intensity, perhaps due to pre-existing fatigue or lowered motivation [52]. Despite being unable to collect RPE data, we firmly believe that exertion would have been similarly high for both trials since the athletes in the present study were of an elite standard and understood that their performances could influence their selection for the national team. To make a pacing error early on in the time trial, when fatigue would have not developed significantly, is perhaps less plausible than a change in the pacing strategy occurring; with this change probably being related to the athletes being in a substantially fatigued, and possibly overreached state during the POST trial. The reduction in the POST trial stroke rate also supports this assertion.

It is conceivable that the positive pacing strategy in the POST trial might indicate the presence of pre-existing fatigue from the four weeks of intensified training. Amann and Dempsey [22] have previously reported a dose-dependent response between the level of pre-exercise fatigue and subsequent pacing strategy during a 5 km cycling trial. They observed a reduction in electrical activation of the vastus lateralis muscle, which coincided with a reduction in power output when participants were pre-fatigued compared to a control condition. It was concluded that a reduction in central motor drive had occurred, which was proportional to the level of pre-existing fatigue. Another potential explanation for the altered pacing strategy in the POST 5 km trial might be related to a decreased energy availability status resulting from a negative energy balance, as discussed earlier. Rauch and colleagues [21] previously demonstrated that glycogen loading coincided with an enhanced power output over a one-hour time trial. The authors postulated that their findings were due to afferent interoceptive feedback informing the insular cortex and prefrontal cortices, and that the resulting glycolytic flux was favourable for maintaining an increased muscle activation (compared to the control trial). It is possible that, in the POST 5 km time trial, a less favourable glycolytic flux was detected by group III/IV afferents (compared to the PRE trial), and subsequently a conscious or sub-conscious decision to reduce central motor drive was initiated, resulting in a loss of boat velocity across the trial. Finally, it is possible that being in a substantially fatigued, and possibly overreached state affected the motivation of the rowers, however as they were elite athletes preparing for a forthcoming Olympic year their level of motivation would likely have been high.

### Wellness and training load monitoring

The decrement in time trial performance and changed pacing strategy in the POST trial, although foreseeable given it occurred at the end of the intensified period of training, reinforces the importance of monitoring training load to ensure adaptation and athlete wellbeing [53]. A number of monitoring techniques have been proposed including external (power output, time-motion analysis), and internal units (heart rate, RPE, BLa and self-reported questionnaires), with dissociation between the two indicative of the fatigue state of the athlete [53]. In the present study, changes in fatigue, vigour, sleep disturbances and total mood disturbance were consistent with an increased training volume, supporting recent research in this area [54–56]. These findings are reinforced by the descriptive information from the weekly training monitoring, which demonstrated slower on-water 1800 m rowing race times and lowered blood lactate concentrations. In addition, the 30-minute rowing ergometer session, at a controlled stroke rate, demonstrated reduced power output. These data along with the increased split times for the POST (vs PRE) on-water rowing 5 km time trial suggests the rowers were experiencing substantial fatigue, and a possibly overreached state after 4 weeks of heavy training. Taken together, these data would indicate that at the end of the training period the athletes suffered either a reduced level of muscle activation or an impairment of force production related to the TMD and reduced energy availability. These findings indicate that a number of parameters (RMR, energy intake, body composition, mood questionnaires, stroke rate and pacing strategy) can be used alongside other validated markers to meaningfully assess responses to intense training periods. Such information, and importantly, monitoring individual changes over time, may provide an early indication of disturbance. This will aid coaches and support staff within the daily training environment to ensure wellbeing and limit unexplained underperformance.

### Limitations

Projects of an applied nature are affected by the logistics of a high performance sport environment, and critically have to accommodate coach and athlete training plans. As a result, it was not possible to obtain a larger sample size or focus on a single sex group of elite rowers in the present study. Therefore whilst care was taken to ensure appropriate scientific rigour in the present study, we acknowledge there remain a number of limitations. Firstly, the authors recognize that conducting RMR measurement in the morning following an afternoon training session is not ideal, but propose that the repeated measurements of RMR in this study were comparable given they were taken under the same conditions. We also note the difficulty in analysing RMR data for a combined sample of male and female athletes; hence the assessment of relative RMR was employed in an attempt to correct for the major gender difference of fat-free mass. RMR values in females may also vary by up to 10% dependent on menstrual status [57]; the specifics of which we were unable to obtain from the present sample. Further, physical characteristics, training and performance data were analysed from the combined sample, which might mean individual differences or responses were overlooked. We acknowledge that the lack of RPE data from the performance trials is not ideal. However, being selection trials with highly motivated, elite athletes, and having been instructed to “complete the distance as quickly as you can”, we are confident that the RPE for both trials would have been similar, and of a maximal exertion. In addition, despite time trials being the most accurate reflection of sport-specific performance [3], it is difficult to standardize conditions in rowing due to environmental influences such as wind speed and direction, currents and water temperature. It is possible that these factors may have influenced the time trial results presented; however we are confident that the normalization of the data minimized the impact of these confounds, and that our analysis is the most practical in the present applied setting. Finally, we acknowledge that future studies of a similar nature should include a follow-up performance trial and psychological measures to elucidate the time course of recovery and whether a state of overreaching truly occurred. This was not possible in our cohort due to travel and competition commitments.

## Conclusion

Heavy periods of training are common during training periodization in an attempt to induce physiological adaptations and improve performance following sufficient recovery. The present study demonstrates, however, that very experienced athletes might not increase energy intake to a sufficient degree to promote optimal adaptation, and suffer ensuing fatigue. We propose a decrease in RMR may be an early indicator of training disturbance, possibly preceded by changes in psychological markers, and that the assessment of changes in exercise regulation and intensity during a time trial may assist in judging the degree of physiological disturbance. Individual responses, however, must be considered, and the present measures may provide additional information to other validated markers of fatigue and potential overreaching. For athletes undertaking similar periods of intensified training, regular monitoring to ensure they consume a sufficient energy intake is vital to supplement the increased training load and promote optimal health. Attaining a greater balance in energy availability would provide more favourable conditions for achieving training consistency, physiological adaptation, and ultimately, performance enhancement.
